# 156. Correlation Between WHO (World Health Organization) Case Definition of Severe Pneumonia and Lung POCUS (Point of Care Ultrasound) vs Chest X-ray (CXR) Findings to Diagnose Pediatric Community-Acquired Pneumonia (CAP) in Limited Resource Settings

**DOI:** 10.1093/ofid/ofab466.156

**Published:** 2021-12-04

**Authors:** Ingrid Y Camelo, Rachel Pieciak, Ilse castro-aragon, Bindu Setty, Lauren Etter, Christopher Gill

**Affiliations:** 1 UMass- Baystate Medical Center/Baystate Children’s Hospital, Northampton, Massachusetts; 2 MPH, Boston, Massachusetts; 3 Boston Medical Center, Chestnut Hill, Massachusetts; 4 Boston University, Boston, Massachusetts; 5 Boston U. School of Public Health, Boston, MA

## Abstract

**Background:**

Childhood pneumonia is one of the leading causes of death in low-income countries. The diagnosis of pediatric pneumonia is a critical epidemiological duty for treatment effectiveness and vaccine surveillance. Previous studies have demonstrated an important lack in correlation between CXR findings and the clinical WHO case definition of severe pneumonia. Lung Point of Care Ultrasound (POCUS) has demonstrated in multiple studies to be more sensitive and specific for diagnosing pneumonia in the pediatric population. With no exposure to radiation, extensive availability in limited-resource settings, and easy interpretation, this modality can be a breakpoint in making a more accurate correlation between pneumonia clinical findings and diagnostic imaging.

**Methods:**

50 children from 1-59 months meeting the WHO case definition of severe pneumonia were enrolled at the Emergency Department at University Teaching Hospital (UTH) in Lusaka, Zambia. Children underwent lung POCUS and CXR. Correlation between symptoms and all abnormalities (consolidation, effusion, and interstitial patterns) seen in both imaging modalities were analyzed by calculating the proportion of children with abnormalities on CXR and ultrasound. Each participant was assigned a score based on findings. 0 = normal, 1 = consolidation only, 2 = Consolidation and non-consolidation (interstitial and/or effusion) and 3 = non-consolidation (interstitial and/or effusion) only.

**Results:**

44 (90%) of children had abnormalities on CXR and 46 (94%) on POCUS. Five children (10%) had normal findings on CXR vs 3 (6%) on Lung POCUS. 4 (8%) had consolidation only on CXR vs 0 (0%) on POCUS. 19 (39%) had consolidation and non-consolidation (interstitial and/or effusion) on CXR vs. 20 (41%) on POCUS. 21 (43%) had non-consolidation (interstitial and/or effusion) only on CXR vs. 26 (53%) on POCUS.

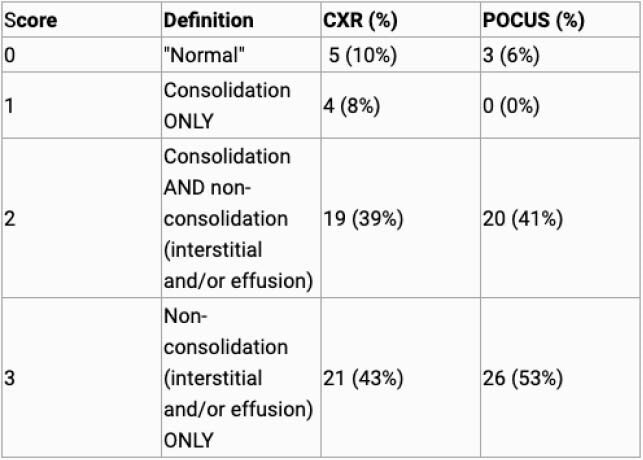

Figure 1. Scores Asigned Based on Imaging Findings for CXR and Lung POCUS

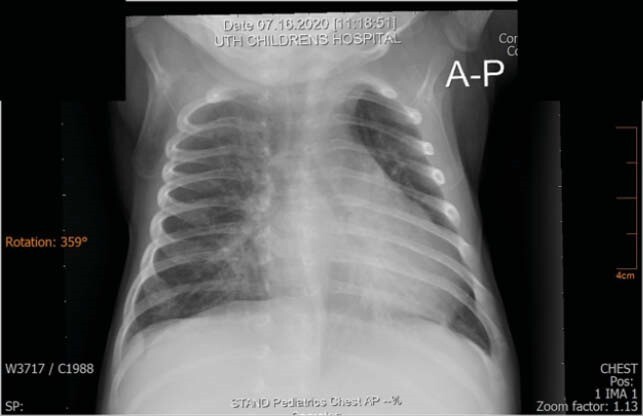

Figure 2. Chest X Ray Anterior Posterior (AP) view showing Bilareral Interstitial Pattern

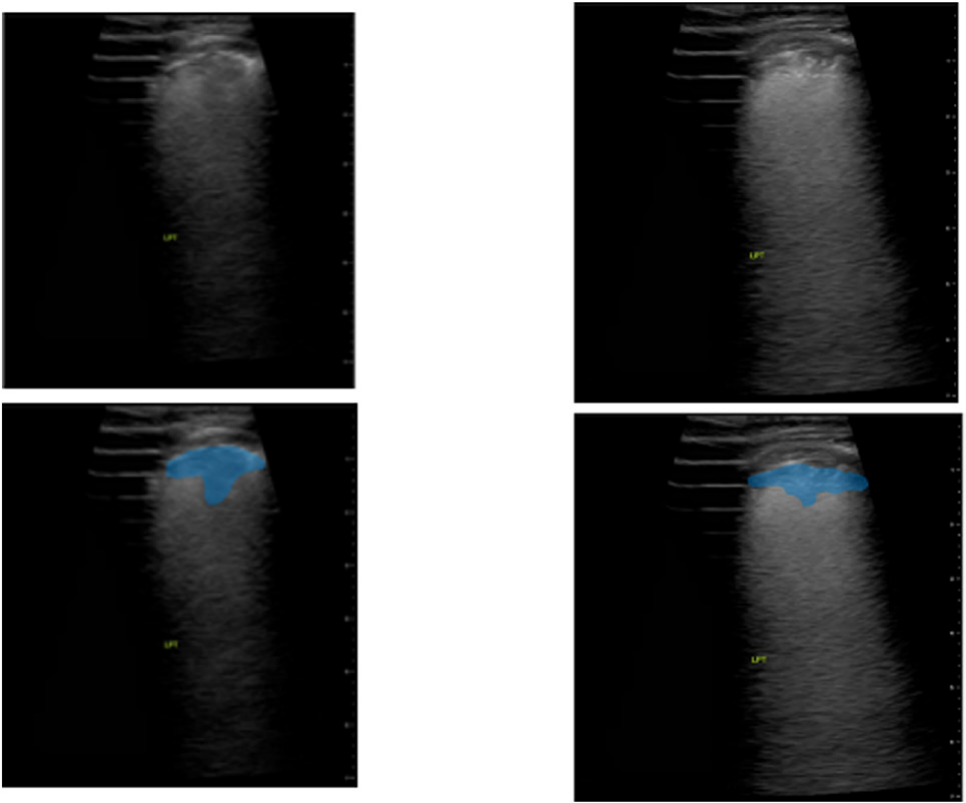

Figure 3. Lung POCUS (Point of Care Ultrasound) findings of bilateral Consolidation and non-consolidation pattern and bilateral interstitial pattern (only finding on CXR)

**Conclusion:**

More children with clinical pneumonia had normal findings on CXR than on POCUS. POCUS was a better imaging technique to show consolidation and non-consolidation patterns than CXR. The higher proportion of children diagnosed with consolidation and non-consolidation patterns on POCUS suggest that CXR might not be the ideal gold standard to diagnose pneumonia in children.

**Disclosures:**

**All Authors**: No reported disclosures

